# Case Report: Cancer spectrum and genetic characteristics of a *de novo* germline *POLD1* p.L606M variant-induced polyposis syndrome

**DOI:** 10.3389/fonc.2023.1222873

**Published:** 2023-09-06

**Authors:** Ying Zhang, Xiaolu Wang, Yuning Zhu, Chong Liang, Lijun Zhao, Qi Meng, Jiani C. Yin, Yuqian Shi, Fufeng Wang, Feng Qin, Ji Xuan

**Affiliations:** ^1^ Department of Pathology, Jinling Hospital, Nanjing University School of Medicine, Nanjing, Jiangsu, China; ^2^ Department of Oncology, The Affiliated Hospital, Nanjing University of Chinese Medicine, Nanjing, Jiangsu, China; ^3^ Department of Neurosurgery Jinling Hospital, Nanjing University School of Medicine, Nanjing, Jiangsu, China; ^4^ Medical Science Liaison, Genetron Health Inc., Beijing, China; ^5^ Geneseeq Research Institute, Nanjing Geneseeq Technology Inc., Nanjing, Jiangsu, China; ^6^ Cancer Center, Jinling Hospital, Nanjing University School of Medicine, Nanjing, China; ^7^ Department of Gastroenterology, Jinling Hospital, Nanjing University, School of Medicine, Nanjing, China

**Keywords:** NGS, POLD1, POLE, germline POLD1 L606M, polyposis syndrome

## Abstract

Germline variations in the DNA polymerase genes, *POLE* and *POLD1*, can lead to a hereditary cancer syndrome that is characterized by frequent gastrointestinal polyposis and multiple primary malignant tumors. However, because of its rare occurrence, this disorder has not been extensively studied. In this report, we present the case of a 22-year-old female patient who had been diagnosed with gastrointestinal polyposis, breast fibroadenoma, multiple primary colorectal cancers, and glioblastoma (grade IV) within a span of 4 years. Next-generation sequencing analysis revealed a germline variant in *POLD1* (c.1816C>A; p.L606M). *In silico* analysis using protein functional predicting software, including SIFT, Polyphen, GERP++, and CADD, further confirmed the pathogenicity of *POLD1* p.L606M (classified as ACMG grade Class 4). In line with polymerase deficiency, both rectal cancer and glioblastoma tissues exhibited a high tumor mutation burden, with 16.9 muts/Mb and 347.1 muts/Mb, respectively. Interestingly, the patient has no family history of cancer, and gene examination of both parents confirms that this is a *de novo* germline variant. Therefore, molecular screening for *POLD1* may be necessary for patients with such a cancer spectrum, regardless of their family history.

## Introduction

DNA replication is a fundamental process for all living organisms, and genes responsible for ensuring the accuracy of DNA replication play vital roles in maintaining health. *POLD1* and *POLE* encode the catalytic subunits of the replicative polymerases, which are key mediators of DNA replication and repair (1–3). Germline variants in *POLD1*/*POLE* can lead to an autosomal dominant hereditary cancer syndrome that predisposes to colorectal polyposis and diverse types of cancer (4–7). Polymerase-associated polyposis syndrome is rare, but it exhibits high penetrance (5, 8–10). In addition, somatic mutations in *POLD1*/*POLE* have also been found in sporadic cancers, emphasizing the significance of DNA replication fidelity in preventing tumorigenesis (8). To date, a variety of germline and somatic *POLD1*/*POLE* alterations have been reported, with p.L424V and p.S478N being the most frequently observed germline variants for *POLE* and *POLD1*, respectively (5, 11). Here, we reported a patient who has experienced symptoms that resembled those of polymerase-associated polyposis syndrome, although with other distinct phenotypes. Genetic testing revealed a *de novo* germline p.L606M variant in *POLD1*.

## Case presentation

**Figure 1:**
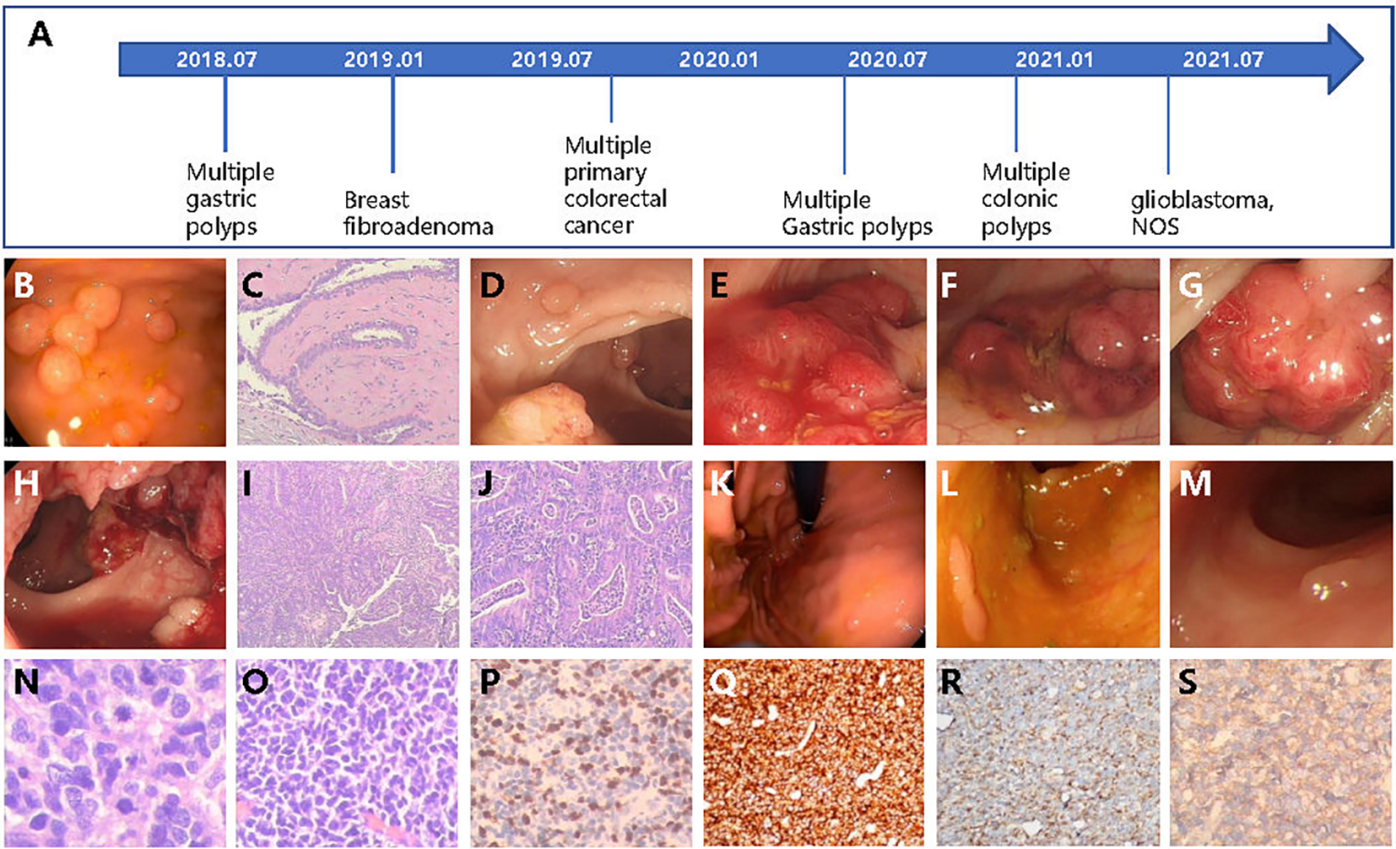
The patient case with multiple cancers or gastrointestinal polyps diagnosed over several years. **(A)** Diagnosis timeline of the patient. **(B)** The multiple gastric polyps found by gastroscope. **(C)** The HE staining of breast fibroadenoma tissue. **(D–H)** The colonoscopy found multiple lesions of cancer and colonic polyps. The HE stain of colonic **(I)** and rectal **(J)** cancer tissues. Newly detected gastric polyps **(K)** found by endoscope and colonic polyps **(L, M)** in 2020. **(N, O)** The HE staining of glioblastoma tissue. **(P–S)** The IHC examination of Ki-67, CD45, Nestin, and β-catenin.

In 2018, a 22-year-old female came to the hospital complaining of intermittent upper abdomen pain and occasional vomiting that had been ongoing for 8 months. An electronic gastroscope examination revealed dozens of polyps in the gastric fundus, body, and horn (**Figures 1A, B**). Subsequently, the patient underwent endoscopic mucosal resection (EMR) to remove all the polyps and experienced relief from her symptoms.

In January 2019, at the age of 23, a lump was discovered in her right breast through routine health check. Subsequently, the lump was removed, and post-operative pathological testing revealed fibroadenoma (**Figures 1A, C**). In the same year, a routine physical examination found multiple space-occupying lesions in the rectum and colon. A further whole abdomen magnetic resonance imaging (MRI) revealed shadows in the rectosigmoid junction, mid-rectum, lower rectum, and proximal anus. Pathological biopsies from the colonoscopy indicated a diagnosis of multiple primary cancers of the colorectum with a stage of IIA (T3N0M0), accompanied by multiple colonic polyposis (**Figure 1A**). To be specific, the relative positions from the anus and the corresponding diagnosis are as follows: 2 cm and 27 cm, low-to-medium differentiated adenocarcinoma; 15 cm, high-grade intraepithelial neoplasia combining partial cancerization; 18 cm, tubulo-papillary structured adenocarcinoma; 30 cm, papillary adenoma; 60 cm, tubular adenoma combining partially mild-to-moderate differentiated atypical hyperplasia; and 65 cm, mixed polyposis (**Figures 1D–J**). The full-body positron emission tomography and computed tomography (PET-CT) as well as brain MRI examination showed no signs of distant metastasis. Therefore, the patient underwent radical surgery resection of the colorectal tumors. Owing to several high recurrent risk factors observed in this patient, such as low-to-medium differentiated cancer and the formation of cancer embolus and multiple primary cancers, the patient received adjuvant therapy (FOLFOX protocol) for 12 cycles from October 2019 to May 2020. Serum tumor markers were monitored after each cycle of adjuvant therapy. Throughout the entire treatment period, all serum markers, including CEA, AFP, CA50, and CA-199, remained within the normal ranges, except for CA125, which was abnormally high during the first three cycles of adjuvant therapy but returned to the normal range afterwards.

The patient has experienced multiple instances of primary colorectal cancers, breast fibroadenoma, and multiple gastric and colonic polyps at a young age, which suggest an inherited disease. Consequently, she was referred to genetic counseling, and an NGS test targeting tumor hot spots of 825 genes was carried out using white blood cell samples. However, no pathogenic or likely pathogenic germline variants were detected at this time.

In 2020, at the age of 24, the patient was re-admitted to the hospital twice in May and December due to the discovery of hundreds of gastric and colonic polyps ranging in size from 0.3 cm × 0.3 cm to 0.6 cm × 0.8 cm (**Figure 1A**). All polyps were removed through either EMR or argon plasma coagulation based on their sizes (**Figures 1K–M**).

In 2021, the patient returned to the hospital, reporting a headache that had persisted for the past week. An enhanced MRI examination of the head revealed an occupation in the right frontal lobe, accompanied by peritumoral brain edema and angiogenesis. Only 1 day after being admitted to the hospital, the patient experienced sudden unconsciousness and right pupil dilation. Hence, with the consent of the guardian, surgical resection of the intracranial tumor was performed. Incision of the frontal cortex showed a blood clot at 2 cm below the cortex, which was surrounded by off-white gelatinous tumor tissue that had a soft texture and clear boundary with the surrounding normal brain tissue, as well as abundant blood supply. The tumor extended to the frontal pole, midline, head of corpus callosum, and front of central anterior gyrus. Post-operative pathological diagnosis indicated glioblastoma, NOS (WHO grade IV, **Figures 1A, N–S**). Subsequently, the patient underwent 30 cycles of adjuvant radiotherapy, followed by oral intake of temozolomide and injection of anlotinib as intensive adjuvant therapy. Currently, the disease is under control, and the patient is in a relatively good condition.

## Identification of germline variants

**Figure 2:**
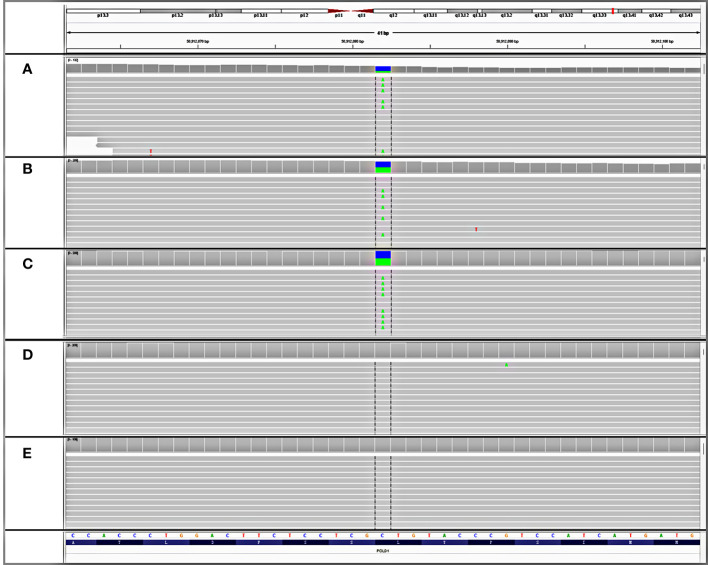
Detection of an unreported *de novo POLD1* L606M germline variant. IGV snapshots of the heterozygous POLD1 variant tested in **(A)** patient’s rectal tissue, **(B)** patient’s glioblastoma tissue, **(C)** patient’s white blood cell, **(D)** father’s white blood cell, and **(E)** mother’s white blood cell.

Although the patient denied a family history of cancer and a previous genetic test failed to detect any detrimental germline variant, the presence of multiple gastrointestinal polyposis and a wide cancer spectrum still raised strong suspicions of hereditary factors. Consequently, the resected rectal tissue, glioblastoma tissue, and white blood cell samples were sent to a CLIA-certified and CAP-accredited central laboratory (Nanjing Geneseeq Technology Inc., Nanjing, China) for additional molecular testing. Next-generation sequencing (NGS) using a targeted panel covering 494 cancer-related genes (detailed gene list in **Supplementary Table S1**) revealed a heterozygous germline variant in the polymerase domain of *POLD1* (c.1816C>A; p.L606M) (**Figures 2A–C**), which had been reported as somatic mutations in ultra-mutated brain, uterine, and endometrial tumors (3, 12–14), although no evidence in clinics of the germline *POLD1* p.L606M has been published yet. The L606 residue in the polymerase domain is highly conserved throughout evolution (15). The yeast analog of this variant, *pol3*-L612M, confers a mutator phenotype with reduced DNA synthesis fidelity (16, 17), which was further confirmed by functional studies in human cells and mouse models (14, 18). Some of the most presumed somatic *POLD1* driver mutations such as p.R689W also map to the polymerase domain (3, 19–21). The pathogenicity of the p.L606M variant was further confirmed by *in silico* analysis using protein functional predicting software, as evidenced by a SIFT score of 0 (deleterious), a PolyPhen score of 0.999 (damaging), a CADD score of 27.6, and a GERP++ RS of 3.62. A total of 304 and 13 somatic variants were detected in the brain tumor and rectal cancer tissues, respectively, with a corresponding tumor mutation burden of 347.1 and 16.9 muts/Mb. Mapping of the substitutions in the brain tumor to the mutational processes from the Catalog of Somatic Mutation in Cancer (COSMIC) database revealed high proportions of SBS15 (0.22), SBS20 (0.19), SDS 14 (0.11), and SBS6 (0.11). All these are predominantly associated with deficient mismatch repair (dMMR) signatures. Notably, SBS20 and SBS14 are also polymerase-associated signatures. Other polymerase-associated signatures, such as SBS 10a/b/c/d and SBS28, were detected at a proportion of less than 0.0001. Signature analysis of the rectal tissue showed high proportions of SBS24 (0.22) and SBS1 (0.18), which are not associated with either dMMR or polymerase signatures. Despite the signature analysis, the affected tissues were microsatellite stable (MSS) as determined by NGS. All these findings are consistent with the diagnosis of germline *POLD1*-induced polyposis syndrome.

To further investigate the origin of this germline variant, NGS tests were performed on the white blood cells of both parents. The *POLD1* germline variant was not detected from either the paternal or maternal side (**Figures 2D, E**). Genetic fingerprint analysis confirmed the parenthood. These findings provide evidence that the *POLD1* germline variant is *de novo*.

After the genetic diagnosis of polymerase-associated polyposis, the patient underwent surveillance and genetic counseling. Given that the disorder is associated with an increased risk of developing various types of cancers, such as colorectal breast, duodenal, ovarian, and central nervous system tumors, the patient was advised to undergo regular colonoscopies and screenings for other cancers.

## Discussion

**Figure 3:**
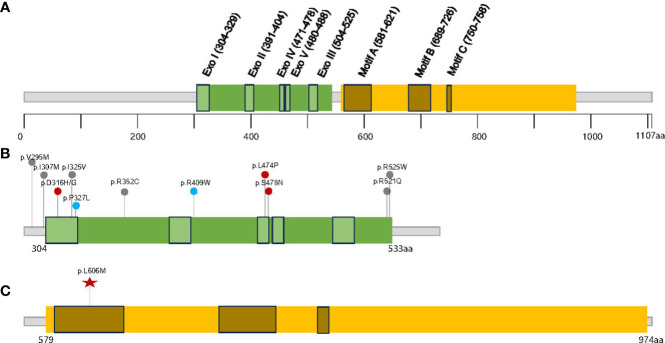
The distribution of the germline POLD1 variants in literature and this study. **(A)** Schematic domain structure and motif sites in human p125/POLD1 (14), The exonuclease and polymerase domains are shown in **(B, C)**, respectively. Red points indicate clearly pathogenic variants, blue points indicate potentially pathogenic variants, and gray points indicate variants with unknown significance; the red star indicates variant detected in this study.

Polymerase-associated polyposis syndrome is an autosomal dominant cancer syndrome caused by pathogenic germline variants in *POLE* and *POLD1*, which predisposes to multiple types of cancer, including CRC, duodenal cancer, and endometrial cancer (22). As this disease is very rare, only a limited number of clinical studies have been reported. In one study, a total of 2,813 unrelated probands were sequenced for *POLE* and *POLD1*, and it was found that polymerase-associated syndrome accounts for 0.1%–0.4% of familial cancers (7). The study also identified five *POLD1* variants in the exonuclease domain, all of which were predicted to be neutral (**Figure 3**). In 2016, Bellido and colleagues reviewed a total number of 529 familial CRC patients and found that among germline *POLD1* variant carriers, the incident rates of CRC, endometrial cancer, breast cancer, and brain tumor were 59.1%, 57.1%, 14.3%, and 4.5%, respectively. The mean ages at diagnosis for CRC and endometrial cancer were 35.9 and 51.4 years old, respectively (6). In another study, 132 patients harboring pathogenic variants in *POLE*/*POLD1* were analyzed, including 105 with *POLE* variants and 27 with *POLD1* variants. Among the *POLD1* carriers, the incidence rates of CRC, endometrial cancer, breast cancer, and brain tumor were 44.4%, 52.9%, 23.5%, and 3.7%, respectively, with a median age at diagnosis of 41, 52, 62, and 26 years old, respectively (22) (**Table 1**).

**Table 1:** The mean or median ages of diagnosis of multiple cancer types in germline POLD1 variant carriers.

Reference	Colorectal cancer	Endometrial cancer	Breast cancer	Brain tumor
Bellido et al., 2016*	35.9	51.4	NA	NA
Palles et al., 2022**	41	52	62	26

*: mean age;

**: median age.

NA, Not Available.

Compared to previous reports, our case has displayed some unique characteristics. Firstly, the patient was diagnosed with breast fibroadenoma, CRC, and glioblastoma in her early twenties, which is earlier than what has been reported in the literature. Secondly, the presence of gastric polyposis, in addition to colonic polyposis, is a novel finding for the disease. Thirdly, this patient has been diagnosed with breast fibroadenoma, which is not typically associated with the cancer spectrum of polymerase-associated polyposis syndrome. We speculated that the difference in the patient’s clinical presentation may be due to the pathogenicity and penetrance of the specific *POLD1* variant, as well as the occurrence of the germline variant through *de novo* formation.

Germline *POLD1*-induced polyposis also displayed a unique spectrum of somatic alterations. Thirteen somatic alterations, including a variant of uncertain significance (VUS) in *APC* p.S1896R, were identified in the rectal tissue. While it is uncertain whether the VUS might lead to loss of function of *APC*, which typically drives the oncogenesis of CRC (23, 24), we propose the *APC* VUS is a *POLD1*-induced variant rather than a driver mutation. In addition, other commonly mutated genes in CRC such as *TP53* and *KRAS* were not detected. In the *POLD1*-deficient glioblastoma tissue, we observed ultra-hypermutation (tumor mutation burden of 347.1 muts/Mb), which is exceedingly rare since glioblastoma is typically classified as a TMB-low tumor type (25). Notably, the presence of the *POLD1* p.L606M variant may be more prevalent in brain tumors. Among the five *POLD1* p.L606M variants identified in the study by Campbell et al. (3), two were found in brain primitive neuroectodermal tumors, and two were found in brain glioblastomas, with a median TMB of 212.4 muts/Mb (range, 51.4–295 muts/Mb). In fact, upon detailed analysis of the *POLD1* drivers in the Campbell et al. study, we found that *POLD1*-driven brain glioblastoma exhibited higher TMB than *POLD1*-driven CRC (median = 228.8 vs. 63.05), although the underlying cause of the tissue-specific difference is unknown. The difference in mutation burden between the rectal and brain tissue may be attributed to variations in the stage of tumor evolution, the distinct microenvironments, and exposure to additional mutational agents in the two tumor types. While both the brain and rectal tissues were MSS, we observed predominantly dMMR-associated signatures in the brain tissue and no polymerase-associated signatures in either lesion, which has also been reported in previous studies on *POLE*/*POLD1* drivers (26). It has also been shown that SBS14 and SBS20 are associated with tumors that exhibit concurrent loss of MMR and polymerase function (27). Importantly, in all cases where the chronological sequence of events could be established, alterations in the polymerase preceded the defects in MMR (27), indicating a dynamic evolution of mutational signatures during cancer development, particularly in tumors with defects in DNA repair. This also suggests that the *POLD1*-altered brain tissue in our case might be at a relatively early stage in its development, transitioning towards acquiring additional MMR defects. Furthermore, other common molecular features of glioblastoma were not detected, such as 1p/19q loss, *EGFR* amplification, or *EGFRvIII* alterations (28). Thus, we hypothesized that cancers induced by germline *POLD1* alterations may exhibit distinct molecular features.

It is also noteworthy that there are some other inherited syndromes that share similar clinical symptoms but with largely distinct molecular landscapes (24, 29, 30). One example is Turcot syndrome, which consistently presents a high risk of central nervous system neoplasms and gastrointestinal cancers paired with intestinal polyps. Studies have revealed that Turcot syndrome is caused by the germline alterations in *APC* or mismatch repair (MMR) genes and often result in microsatellite-instable (MSI) tumors (29, 31). On the other hand, hereditary colorectal cancers can manifest as both polyposis and nonpolyposis conditions (32). The most common inherited disease that predisposes individuals to colorectal and endometrial cancers is Lynch syndrome, which is a non-polyposis condition caused by pathogenic germline variants in the MMR genes that results in MSI. By contrast, tumors associated with polymerase-associated polyposis syndrome are almost always MSS. In our case, even though multiple variants were detected in MMR genes, including *MLH1*, *MLH3*, *MSH2*, *MSH4*, and *MSH6*, the tumor tissues were MSS. This finding is consistent with the typical manifestation of polymerase-associated polyposis syndrome. Accumulation of these MMR gene variants were likely a consequence of *POLD1*-induced hypermutation. Other polyposis conditions often exhibit overlapping and distinct cancer spectra. For example, familial adenomatous polyposis, caused by pathogenic germline variants in *APC*, is characterized by the presence of hundreds to thousands of adenomatous colonic polyps. *MUTYH*-associated polyposis is an autosomal recessive disorder that involves biallelic germline variants in *MUTYH*. Affected individuals tend to develop multiple adenomatous colonic polyps, along with an increased risk for small-bowel polyposis. Many other genetic disorders also result in an increased risk of colorectal cancer, including Li-Fraumeni syndrome (TP53), juvenile polyposis syndrome (SMAD4 and BMPR1A), Peutz–Jeghers syndrome (STK11), and Cowden syndrome (PTEN) (32, 33). Therefore, a high-throughput genetic examination can aid in accurately distinguishing these diseases, and cancer surveillance is essential for patients with these conditions after the genetic diagnosis.

In summary, our case reported a *de novo* germline *POLD1* p.L606M variant that led to early-onset multiple types of cancer, coupling with multiple gastrointestinal polyposis. NGS targeting the whole exome of relevant genes could facilitate an accurate diagnosis of suspected hereditary diseases.

## Methods

### Next-generation sequencing

Sample processing and sequencing were performed in a CLIA-certified and CAP-accredited laboratory (Geneseeq Technology Inc., Nanjing, China) as previously described (34). In brief, genomic DNAs from FFPE sections and the whole blood control samples were extracted with the QIAamp DNA FFPE Tissue Kit and the DNeasy Blood and tissue kit (Qiagen), respectively, and quantified by Qubit 3.0 using the dsDNA HS Assay Kit (ThermoFisher Scientific). Library preparations were performed with the KAPA Hyper Prep Kit (KAPA Biosystems). Customized xGen lockdown probes (Integrated DNA Technologies) targeting whole exons of 494 cancer-relevant genes (**Supplementary Table S1**) were used for hybridization enrichment. The capture reaction was performed with Dynabeads M-270 (Life Technologies) and xGen Lockdown hybridization and wash kit (Integrated DNA Technologies) according to the manufacturers’ protocols. Libraries were on-beads PCR-amplified, purified, sized and quantified, and sequenced on an Illumina HiSeq4000 platform.

### Mutation calling

Trimmomatic was used for FASTQ file quality control. Leading/trailing low quality (quality reading below 20) or N bases were removed. Paired-end reads were then aligned to the reference human genome (build hg19), using the Burrows–Wheeler Aligner (BWA) with the parameters. PCR deduplication was performed using Picard and local realignment around indels, and base quality score recalibration was performed using GATK3. Furthermore, samples with a mean dedup depth <30× were removed. Cross-sample contamination was estimated using ContEst (Broad Institute). Germline and somatic mutations were called using the GATK haplotypecaller and Mutect2, respectively. Tumor mutation burden was counted by summing all base substitutions and indels in the coding region of targeted genes, including synonymous alterations to reduce sampling noise and excluding known driver mutations as they were over-represented in the panel, as previously described (34, 35).

## Data availability statement

The data presented in the study are deposited in the Genome Sequence Archive (Genomics, Proteomics & Bioinformatics 2021) in National Genomics Data Center (Nucleic Acids Res 2022), China National Center for Bioinformation/Beijing Institute of Genomics, Chinese Academy of Sciences (GSA-Human), publicly accessible at https://ngdc.cncb.ac.cn/gsa-human, accession number HRA005282.

## Ethics statement

Written informed consent was obtained from the individual(s) for the publication of any potentially identifiable images or data included in this article.

## Author contributions

YZ, FQ, JX, and XW: Conception, design, and manuscript review. YNZ and JX: Material preparation. CL and LZ: Literature research and discussion. QM, FW, JY, and YS: Analysis of original data and bio-informatic research. All the authors agreed to the submission of the version.

## Supplementary material

Supplementary Table 1 Detailed gene list in 494 panels.
