# Supplementary material for: Case Report: Cancer spectrum and genetic characteristics of a de novo germline POLD1 p.L606M variant-induced polyposis syndrome
Source: Front Oncol. 2023 Sep 6;13:1222873. doi: 10.3389/fonc.2023.1222873 (PMC10516538; doi:10.3389/fonc.2023.1222873)
Supplement: Supplementary Table 1 — Detailed gene list in 494 panels. [file Table_1.docx]

**Table s1. Detailed gene list in 494 panel.**

| 1p/19q | CCNE1 | DTL(CDT2) | GATA2 | KIT | NOTCH3 | PTCH2 | SOS1 |
| --- | --- | --- | --- | --- | --- | --- | --- |
| ABCB1 (MDR1) | CD274 (PD-L1) | DUSP2 | GATA3 | KITLG | NPM1 | PTEN | SOX2 |
| ABCC2 (MRP2) | CD74 | EGFR | GATA4 | KLLN | NQO1 | PTK2 | SPOP |
| ACVR1 | CDA | EIF1AX | GATA6 | KMT2A (MLL) | NRAS | PTPN11 | SPRED1 |
| ADGRB3 (BAI3) | CDC73 | EML4 | GFAP | KMT2B | NRG1 | PTPN13 | SPRY4 |
| ADH1B | CDH1 | EMSY (c11orf30) | GFI1 | KMT2C (MLL3) | NSD1 | PTPRD | SRC |
| AFDN (MLLT4) | CDK10 | EP300 | GFI1B | KMT2D (MLL2) | NTHL1 | PTPRZ1 | SRSF2 |
| AIP | CDK12 | EPAS1 | GLI1 | KRAS | NTRK1 | QKI | SRY |
| AKT1 | CDK4 | EPCAM | GLI2 | LDB1 | NTRK2 | RAC1 | STAG2 |
| AKT2 | CDK6 | EPHA2 | GNA11 | LHCGR | NTRK3 | RAC3 | STAT3 |
| AKT3 | CDK8 | EPHA3 | GNAQ | LMO1 | NUTM1 | RAD50 | STK11 |
| ALDH2 | CDKN1A | EPHA5 | GNAS | LRP1B | OLIG2 | RAD51 | STMN1 |
| ALK | CDKN1B | ERBB2 (HER2) | GPS2 | LYN | OTX2 | RAD51B | SUFU |
| AMER1 (FAM123B) | CDKN1C | ERBB3 | GREB1 | LZTR1 | PAK3 | RAD51C | TACC3 |
| APC | CDKN2A | ERBB4 | GREM1 | MAP2K1 (MEK1) | PALB2 | RAD51D | TAP1 |
| AR | CDKN2B | ERBIN (ERBB2IP) | GRIN2A | MAP2K2 (MEK2) | PALLD | RAD54L | TAP2 |
| ARAF | CDKN2B-AS1 | ERCC1 | GRM3 | MAP2K4 | PARP1 | RAF1 | TCF12 |
| ARID1A | CDKN2C | ERCC2 | GRM8 | MAP3K1 | PARP2 | RARA | TCF4 |
| ARID1B | CEBPA | ERCC3 | GSE1 | MAP3K4 | PATZ1 | RARG | TEK |
| ARID2 | CEP57 | ERCC4 | GSTM1 | MAX | PAX5 | RASGEF1A | TEKT4 |
| ARID5B | CHD4 | ERCC5 | GSTM4 | MC1R | PBRM1 | RB1 | TERC |
| ASCL4 | CHD7 | ESR1 | GSTP1 | MCL1 | PDCD1 (PD1) | RECQL4 | TERT |
| ASXL1 | CHD8 | ETV1 | GSTT1 | MDM2 | PDCD1LG2 (PD-L2) | RELA | TET2 |
| ATF1 | CHEK1 | ETV4 | H3F3A | MDM4 | PDE11A | RELN | TGFBR2 |
| ATIC | CHEK2 | ETV5 | HDAC2 | MECOM | PDGFRA | RET | THADA |
| ATM | CIC | ETV6 | HDAC9 | MED12 | PDGFRB | RHBDF2 | TMEM127 |
| ATR | CREBBP | EWSR1 | HGF | MEF2B | PDK1 | RHOA | TMPRSS2 |
| ATRX | CRKL | EXT1 | HIST1H3B | MEN1 | PGR | RICTOR | TNFAIP3 |
| AURKA | CSF1R | EXT2 | HIST1H3C | MET | PHLDB1 | RNF43 | TNFRSF11A |
| AURKB | CSNK2B | EZH2 | HLA-A | MGMT | PHOX2B | ROS1 | TNFRSF14 |
| AXIN2 | CTCF | EZR | HNF1A | MITF | PIK3C3 | RPTOR | TNFRSF19 |
| AXL | CTDNEP1 | FAM131B | HNF1B | MLH1 | PIK3CA | RRAS2 | TNFSF11 |
| B2M | CTLA4 | FANCA | HOXB13 | MLH3 | PIK3CD | RRM1 | TOP1 |
| BAD | CTNNB1 | FANCC | HRAS | MLLT1 | PIK3R1 | RTEL1 | TOP2A |
| BAK1 | CUL3 | FANCD2 | IDH1 | MLLT3 | PIK3R2 | RUNX1 | TP53 |
| BAP1 | CUX1 | FANCE | IDH2 | MN1 | PKHD1 | RUNX1T1 | TP63 |
| BARD1 | CXCR4 | FANCF | IFNA6 | MPL | PLAG1 | SBDS | TPMT |
| BAX | CXX5 | FANCG | IFNB1 | MRE11 (MRE11A) | PLCB4 | SDC4 | TSC1 |
| BCL2 | CYLD | FANCI | IFNE | MSH2 | PLK1 | SDHA | TSC2 |
| BCL2L11 (BIM) | CYP19A1 | FANCL | IFNG | MSH6 | PMS1 | SDHB | TSHR |
| BCOR | CYP2A13 | FANCM | IFNGR1 | MTAP | PMS2 | SDHC | TTF1 |
| BCORL1 | CYP2A6 | FAT1 | IFNGR2 | MTHFR | POLD1 | SDHD | TUBB3 |
| BCR | CYP2A7 | FBXW7 | IGF1R | MTOR | POLD3 | SEPTIN9 (SEPT9) | TYMS |
| BIRC3 | CYP2B6*6 | FGF19 | IGF2 | MUTYH | POLE | SETBP1 | U2AF1 |
| BLM | CYP2C19*2 | FGFR1 | IKBKE | MYB | POLH | SETD2 | UGT1A1 |
| BMPR1A | CYP2C9*3 | FGFR2 | IKZF1 | MYBL1 | POT1 | SF3B1 | VAMP2 |
| BRAF | CYP2D6 | FGFR3 | IL7R | MYC | PPARD | SGK1 | VEGFA |
| BRCA1 | CYP3A4*4 | FGFR4 | INPP4B | MYCL (MYCL1) | PPM1D | SKP2 | VHL |
| BRCA2 | CYP3A5 | FH | IRF2 | MYCN | PPP2R1A | SLC34A2 | WAS |
| BRD4 | CYSLTR2 | FLCN | JAK1 | MYD88 | PPP2R2A | SLC3A2 | WRN |
| BRIP1 | DAXX | FLT1 (VEGFR1) | JAK2 | MYH9 | PRDM1 | SMAD2 | WT1 |
| BTG2 | DDR2 | FLT3 | JAK3 | NAT1 | PRDM6 | SMAD3 | XPA |
| BTK | DDX31 | FLT4 | JARID2 | NBN | PREX2 | SMAD4 | XPC |
| BUB1B | DDX3X | FOXA1 | JUN | NCOR1 | PRF1 | SMAD7 | XRCC1 |
| CASP8 | DENND1A | FOXL2 | KBTBD4 | NCOR2 | PRKACA | SMARCA2 | XRCC2 |
| CBL | DHFR | FOXO1 | KDM4C | NF1 | PRKAR1A | SMARCA4 | YAP1 |
| CBLB | DICER1 | FOXP1 | KDM5A | NF2 | PRKCI | SMARCB1 | ZIC1 |
| CCDC26 | DKK1 | FOXR2 | KDM6A | NFE2L2 | PRKDC | SMARCC2 | ZMYM3 |
| CCN6 (WISP3) | DLL3 | FRG1 | KDR (VEGFR2) | NFKBIA | PRKN (PARK2) | SMARCD2 | ZNF217 |
| CCND1 | DNMT3A | FUBP1 | KEAP1 | NKX2-1 | PRSS1 | SMO | ZNF703 |
| CCND2 | DOT1L | GABRG1 | KIAA1549 | NOTCH1 | PRSS3 | SNCAIP |  |
| CCND3 | DPYD | GATA1 | KIF1B | NOTCH2 | PTCH1 | SOCS1 |  |
